# Activator of G-protein Signaling 3 Controls Renal Epithelial Cell Survival and ERK5 Activation

**DOI:** 10.5334/1750-2187-10-5

**Published:** 2015-11-27

**Authors:** Shauna A. Rasmussen, Michelle Kwon, Jeffrey D. Pressly, Joe B. Blumer, Kevin R. Regner, Frank Park

**Affiliations:** Medical College of Wisconsin, US; University of Tennessee Health Science Center, College of Pharmacy, Department of Pharmaceutical Sciences, Memphis, TN 38104, US; Medical University of South Carolina, Department of Cell and Molecular Pharmacology and Experimental Therapeutics, Charleston, SC

**Keywords:** Activator of G-protein Signaling 3, cell signaling, renal epithelial cell, lentiviral vectors

## Abstract

Activator of G-protein signaling 3 (AGS3) is an accessory protein that functions to regulate the activation status of heterotrimeric G-protein subunits. To date, however, the downstream signaling pathways regulated by AGS3 remain to be fully elucidated, particularly in renal epithelial cells. In the present study, normal rat kidney (NRK-52E) proximal tubular epithelial cells were genetically modified to regulate the expression of AGS3 to investigate its role on MAPK and mTOR signaling to control epithelial cell number. Knockdown of endogenous AGS3 protein was associated with a reduced phosphorylated form of ERK5 and increased apoptosis as determined by elevated cleaved caspase-3. In the presence of the ERK5 inhibitor, BIX02189, a significant 2-fold change (P < 0.05) in G2/M transition state was detected compared to control conditions. Neither of the other MAPK, ERK1/2 or p38 MAPK, nor another pro-survival pathway, mTOR, was significantly altered by the changes in AGS3 protein levels in the renal epithelial cells. The selective ERK5 inhibitor, BIX02189, was found to dose-dependently reduce NRK cell number by up to 41% (P < 0.05) compared to control cells. In summary, these findings demonstrated that cell viability was regulated by AGS3 and was associated with ERK5 activation in renal epithelial cells.

## Introduction

Accessory proteins are involved in the regulation of signal processing through their unique interactions with individual subunits in the heterotrimeric G-protein complex [1]. Accessory proteins have the ability to control the strength, efficiency, time of activation, and specificity of the signaling output. Activators of G-protein Signaling (AGS) are a group of accessory proteins that were isolated from genetically modified yeast to identify cDNAs that could activate G-proteins in the absence of a cell surface receptor [2,3]. AGS proteins are classified in four distinct categories due to their diverse modes of action to modulate individual subunits from heterotrimeric G-proteins [4]. Specifically, AGS proteins have the capability to influence the dynamics of guanine nucleotide binding, hydrolysis and dissociation of Ga subunits during the G-protein activation-deactivation cycle. In addition, AGS proteins can alter the stability and availability of individual Gα and Gβγ subunits to control the rate of formation for the heterotrimeric Gαβγ. In some cases, this can affect the ability of cell surface G-protein coupled receptors to transmit extracellular signals into the cell [5,6,7].

One of the first proteins identified as an AGS protein was Activator of G-protein Signaling 3 (AGS3) also known as G-protein signaling modulator 1 (GPSM1) [2,3]. Under normal conditions, the highest level of AGS3 mRNA and protein expression was initially detected in distinct regions within the brain [8,9] and heart [8,9,10] from mice and rats. In the past few years, however, there are increasing numbers of studies demonstrating the expression and biological role of AGS3 in various other mammalian tissues [11,12,13,14,15,16]. In the kidney, robust AGS3 expression could be detected in actively dividing proximal tubular epithelial cells during the recovery phase following acute kidney injury [13,15] or in cystic collecting duct epithelial cells during polycystic kidney disease [12]. To date, the signaling mechanisms controlled by AGS3 in the tubular epithelial cell during normal and varying states of injury or disease remains to be fully elucidated. The C-terminal region of AGS3 has G-protein regulatory (GPR) motifs, which have the ability to selectively interact with Gα_i/o_ bound to GDP [17]. Subsequently, the AGS3-Gα_i/o_ complex can regulate downstream signaling by either preventing the inhibitory actions on adenylyl cyclase [18,19], or block the re-association with its native partner, Gβγ, to activate signaling pathways dependent upon the increased pools of Gβγ dimers [20,21]. In renal epithelial cells, AGS3-mediated Gβγ-dependent signaling was shown to be associated with regulating cell number [12,14,15,22]. Although several modes of action can be triggered by AGS3-mediated Gβγ signaling, such as regulation of mitotic spindle orientation [20], polycystin PC1/PC2 channel activity [12], adenylyl cyclase activity [18] and leukocyte chemotaxis [11], their roles in tubular epithelial cell survival have yet to be fully explored.

There is also increasing evidence that Gβγ can activate a multitude of other signaling pathways involved in cell survival and proliferation, including mammalian target of rapamycin (mTOR) [23] and mitogen activated protein kinases (MAPK) [24,25]. Because of the potential role for MAPK in various renal pathologies, including tubular recovery following ischemia-reperfusion injury and cystic disease pathogenesis, the present study was designed to focus on the MAPK pathways, which include the following kinases, extracellular regulated signaling kinase 1 and 2 (ERK1/2), SAPK/JNK, p38 MAPK and ERK5/big mitogen activated-protein kinase 1 (BMK1) [25,26], to investigate whether the changes in the endogenous expression of AGS3 and its associated effects on tubular epithelial cell number are attributed to alterations in either of the mTOR or MAPK signaling pathways.

## Materials and Methods

**Lentiviral vector production and genetic modification of renal epithelial cells.** Replication-defective lentiviral vectors expressing control and AGS3-specific shRNA were generated as previously described [12,14,15,27]. Human full-length *AGS3* cDNA was RT-PCR amplified from total RNA isolated from human embryonic kidney (HEK) 293T cells, and TA cloned into pCR2.1 (Life Technologies, Carlsbad, CA). The human *AGS3* cDNA fragment was sub-cloned into pHR(+).cUb.MCS.R(-)W(+) to make pHR(+).cUb.h*AGS3*.R(-)W(+) using standard molecular techniques.

Co-transfection of the lentiviral transfer, packaging and envelope plasmids was performed into human embryonic kidney 293T cells (5–6 x 10^6^ cells in 10-cm plates) to generate the lentiviral vector particles as previously described [12,14,15,28,29]. After 48 hours, the conditioned media containing the lentiviral vectors was collected, and serially transduced into NRK cells. At this point, the cells were expanded for cell counting [12,14,15] or protein lysates were generated for immunoblot analysis as previously described [12,14,15,28,29].

**Cell counting.** NRK cells modified with lentiviral vectors were plated into 6-well dishes using low serum (1% FBS) in DMEM supplemented with penicillin, streptomycin and glutamine. The following day (18–24 hours later), the cells were counted to confirm that the starting number of cells were within close range of each other. Generally, the starting number of cells ranged between 30,000–50,000 cells/well as determined by hemocytometry [14,15], and this was considered the starting (t = 0 hr) time point. Afterwards, the cells were counted every 24 hours for a 2–3 day period in the presence and absence of the following drugs: BIX02189 (ERK5 inhibitor; 3 and 10 μM; Selleck Chemicals), or everolimus (mTOR inhibitor; 3 and 10 nM; Selleck Chemicals). As a negative control, the appropriate vehicle solution was incubated with the cells. Each cell line was counted in triplicate or more each day, and the data were graphed to demonstrate the cell numbers for each day.

**Antibodies.** phospho-ERK1/2 (cat #9101), total ERK1/2 (cat #9102), phospho-p38 MAPK (ct #9211), total p38 MAPK (cat #9212), and phospho-ERK5 (cat #3371), total ERK5 (cat #3372), phospho-mTOR (Ser2481) (cat #2974), phospho-mTOR (Ser2448) (cat #2971), total mTOR (cat #2983), and cleaved caspase-3 (cat #9661) were obtained from Cell Signaling (Danvers, MA). β-actin or GAPDH were obtained from Sigma (St. Louis, MO).

**Western blot analysis and band intensity analysis.** Protein lysates were isolated from the cells using 1X RIPA buffer containing protease (Roche) and phosphatase inhibitors (Pierce) for Western blot analysis as previously described by our lab [12,14,15,28,29]. In brief, 30–50 μg of protein were size separated using a 4–20% SDS-PAGE gel, and transferred onto PVDF membrane. The membranes were washed in TBS-T, and primary antibodies were incubated for either 2–4 hours at room temperature or overnight at 4°C. The membranes were washed in TBS-T, and the appropriate secondary antibody was added (1:1,000–1:2,000 dilution) for 2 hours at room temperature. The membranes were washed, placed in chemiluminescent solution (Amersham), and exposed to film. Band intensities for specific signaling and housekeeping proteins were determined by NIH ImageJ.

**Cell cycle analysis.** NRK-SCA cells were plated on 10-cm plates and allowed to propagate using low serum (1%) DMEM supplemented with penicillin, streptomycin, and glutamine. Upon reaching 70–80% confluence, BIX-02189 (10 µM) or vehicle (DMSO) was added to the cells overnight (16–20 hrs). At this point, the cells were collected by trypsinization, several washes in phosphate-buffer saline (pH 7.4), and then resuspended in propidium iodide (PI) buffer (containing 20 µg/mL RNase (Life Technologies), 0.1% Tween-20, and 50 µg/mL propidium iodide). The cells were counted using a LSRII flow cytometer (Becton Dickinson), and the PI-positive cells were separated using the BD FACSDiva software. Subsequently, univariate analysis was performed on the raw data to assess the various states of the cell cycle (ModFit LT version 3.0; Verity Software).

**Statistical analysis.** All values are shown as mean ± SEM. Unpaired student’s t-test or one-way ANOVA was used to compare the differences between experimental groups for densitometry. Differences in cell number were calculated with one-way ANOVA, and Tukey post-hoc analysis was performed if significant difference was determined between groups. All statistical analyses were performed using Prism 6.0 (GraphPad, La Jolla, CA).

## Results

**Figure 1:**
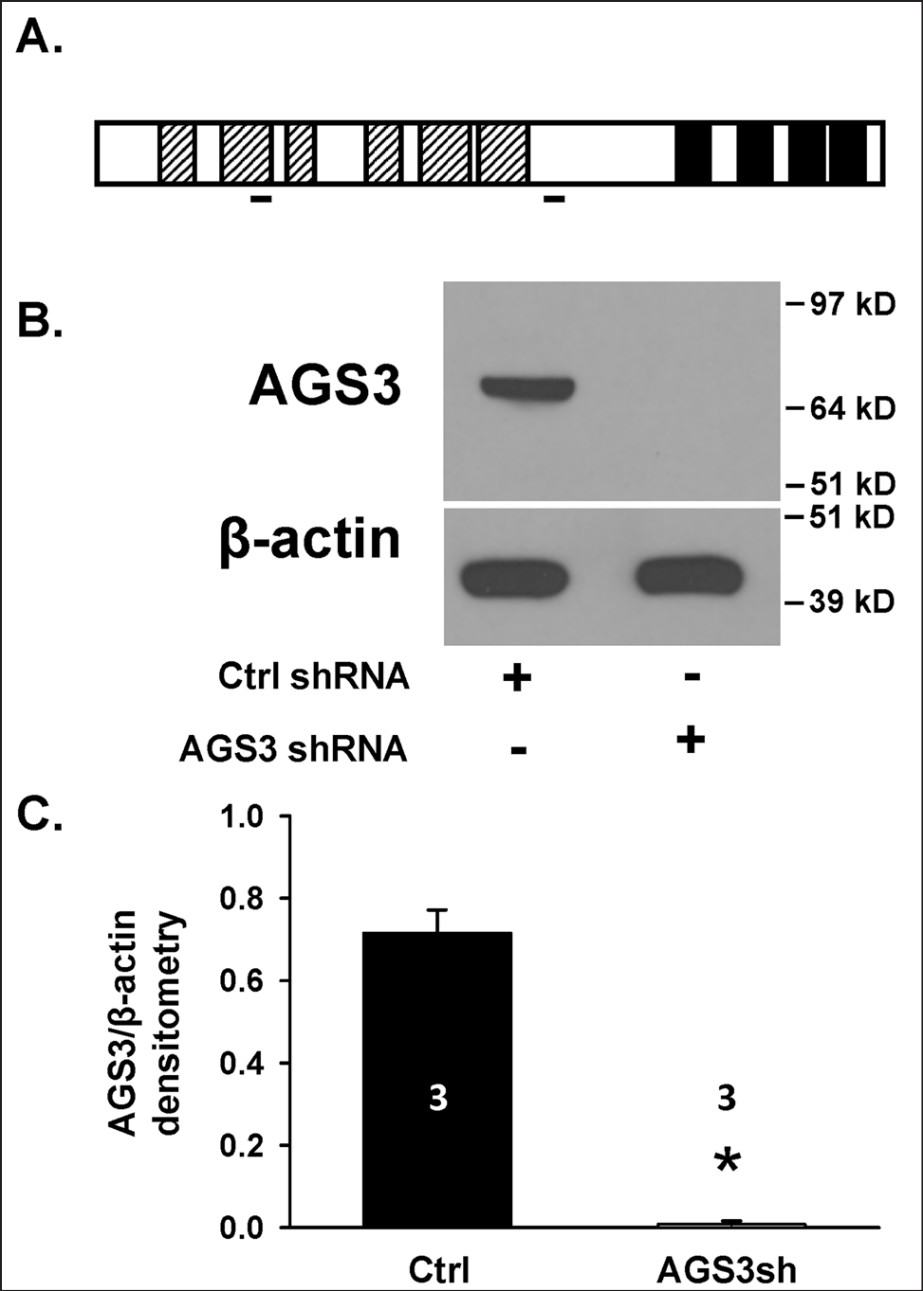
**Genetic modification of NRK epithelial cells using lentiviral vectors to reduce endogenous levels of AGS3.** (A) Schematic of AGS3 protein structure. Hatched bars = TPR motifs; solid black = G-protein regulatory (GPR) domains; solid lines = AGS3 shRNA target sites. (B) Protein lysates from NRK-52E cells transduced with lentiviral vectors expressing either control (NRK-Ctrl) or two distinct AGS3-specific shRNA (NRK-AGS3sh) were isolated for immunoblot analysis using a polyclonal AGS3 antibody. β-actin was shown as a loading control. Protein standards are shown on the right (in kD) for each blot image. (C) Graphical analysis of the AGS3 band intensity. Three different samples were used in each group, and were shown in the bars. * P < 0.001 significant difference between NRK cells expressing Ctrl versus AGS3-specific shRNA.

**Effect of reducing endogenous AGS3 protein expression on cell number.** Genetically modified normal rat NRK-52E renal epithelial cells using a combination of two distinct AGS3 short hairpin RNAs (see Figure 1A for the target location) were generated to evaluate changes in signal transduction pathways and cell growth compared to control NRK cells. The AGS3-deficient NRK cells were denoted as NRK-AGS3sh and the endogenous AGS3 protein levels (P < 0.05; Figure 1B and 1C) were shown to be completely reduced. NRK cells transduced with a control shRNA (NRK-Ctrl) were used as a comparative cell line to demonstrate that the effect of lentiviral vector integration had no impact on AGS3 expression or normal cell growth. The validation method in which we chose a combination of two AGS3-specific shRNA to reduce the endogenous levels of AGS3 was previously published by our lab [14].

**Figure 2:**
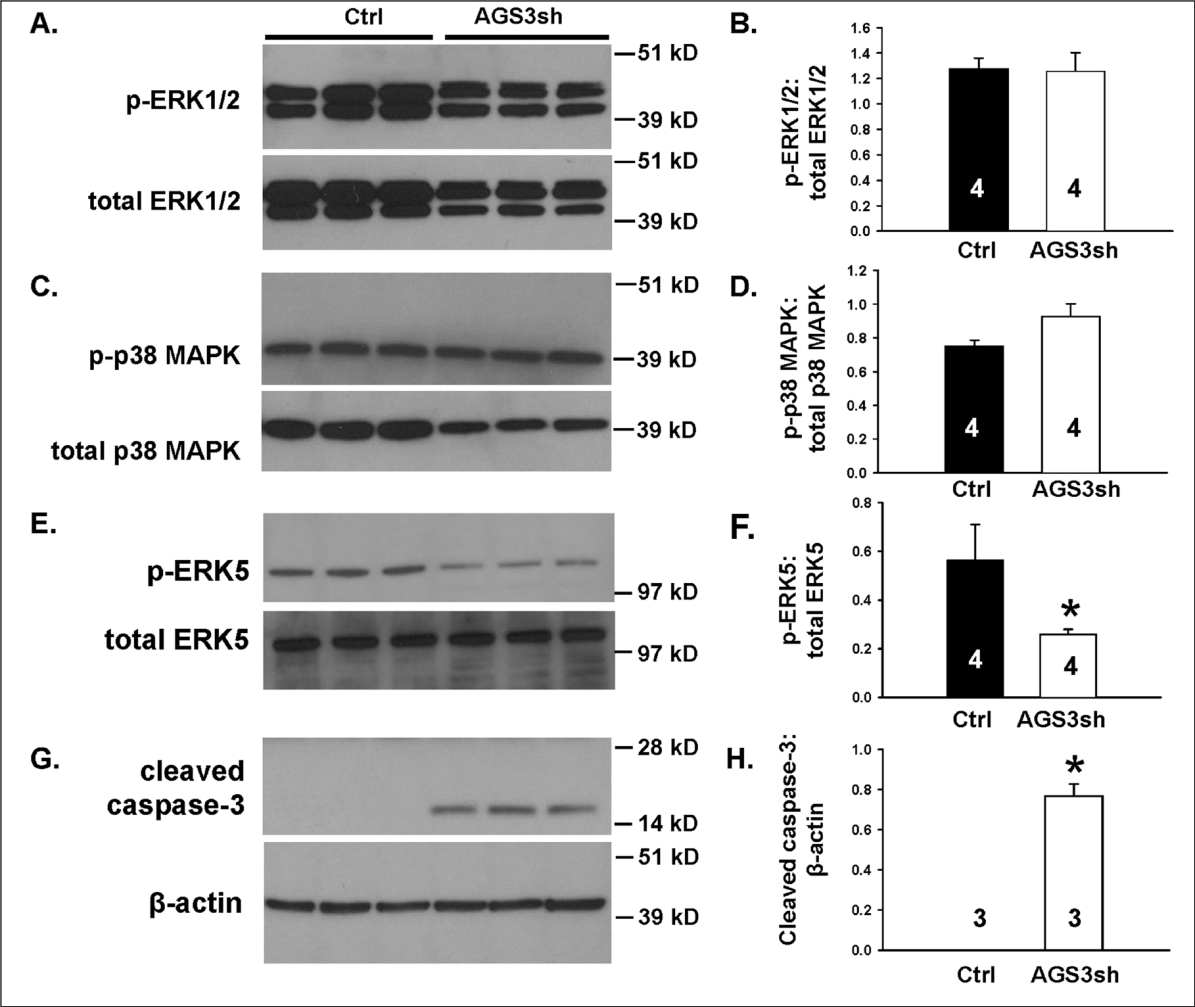
**Reduction in endogenous AGS3 protein by short hairpin RNA technology decreases ERK5 activation and promotes cleavage of caspase-3.** NRK cells transduced with lentiviral vectors expressing either control (Ctrl) or two distinct AGS3-specific shRNA (AGS3sh) were plated and collected after 2–3 days in culture for protein lysate isolation. Phospho- and total (A) ERK1/2, (C) p38 MAPK, (E) ERK5, and (G) cleaved caspase-3 were detected by Western blot analysis. β-actin was used as a loading control in (G). Protein standards are shown on the right (in kD) for each blot image. Densitometry of the bands was determined for each of the MAPK and cleaved caspase-3 using NIH ImageJ software. Ratios of the phospho-to-total bands for the MAPK (B, D, F) or cleaved caspase-3:β-actin (H) were calculated and graphed. Sample numbers were shown in the bars.

In our initial set of experiments, reductions in endogenous AGS3 in NRK cells did not significantly (P > 0.05) alter the ratio of phospho-to-total ERK1/2 (Figure 2A and 2B) or p38 MAPK (Figure 2C and 2D). However, there was a significant reduction (P < 0.05) in the phosphorylated form of ERK5 compared to the total ERK5 (Figure 2E and 2F).

**Figure 3:**
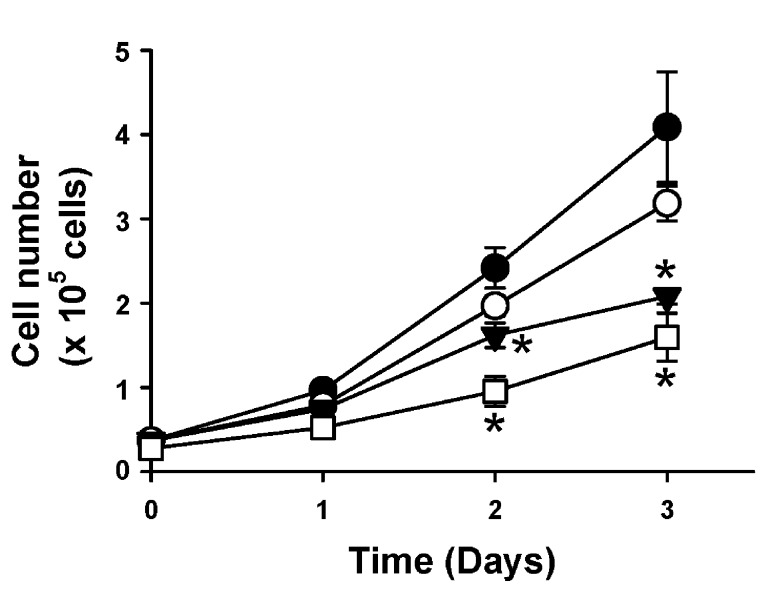
**ERK5 inhibition reduces renal epithelial cell number.** NRK cells transduced with lentiviral vectors expressing either control (Ctrl) or two distinct AGS3-specific shRNA (AGS3sh) were plated. NRK-Ctrl cells were counted in the presence of either vehicle (DMSO; ●), or two different doses of a selective ERK5/BMK1 inhibitor, BIX02189, at 3 (○) and 10 µM (▼). As a comparison, cell counts were determined for the NRK-cells expressing the AGS3 shRNA (NRK-AGS3sh; □). *P < 0.05 significant difference between vehicle (DMSO)-treated group. n = 3–6 experiments per time point.

To confirm whether selective inhibition of ERK5 activity would reduce cell numbers, genetically modified NRK cells expressing either control (scrambled) or AGS3 shRNA were counted in the presence and absence of a selective ERK5 inhibitor, BIX02189, at 3 and 10 μM over a 72 hour period (n = 3–6 values per time point; Figure 3). Immunoblot analysis using protein lysates from NRK-Ctrl cells treated with increasing doses of BIX02189 (1.5–10 μM) demonstrated reduced phosphorylation of ERK5, but not ERK1/2, compared to vehicle (DMSO)-treated cells (**Supplemental Figure 1**). In the presence of two different doses of BIX02189 within the range used for the immunoblot, a dose-dependent reduction in cell number was calculated at both doses, but reached significance at the higher dose (10 μM; P < 0.05 at both time points) compared to the vehicle-treated NRK-Ctrl cells. Consistent with previous experiments in our lab [14,15,22], NRK-AGS3sh cells expanded significantly slower than NRK-Ctrl cells through the experimental period (Figure 3). Treatment of the NRK cells expressing AGS3 shRNA with BIX02189 resulted in poor viability of the cells that could not be maintained beyond 24 hours of incubation (data not shown).

**Figure 4:**
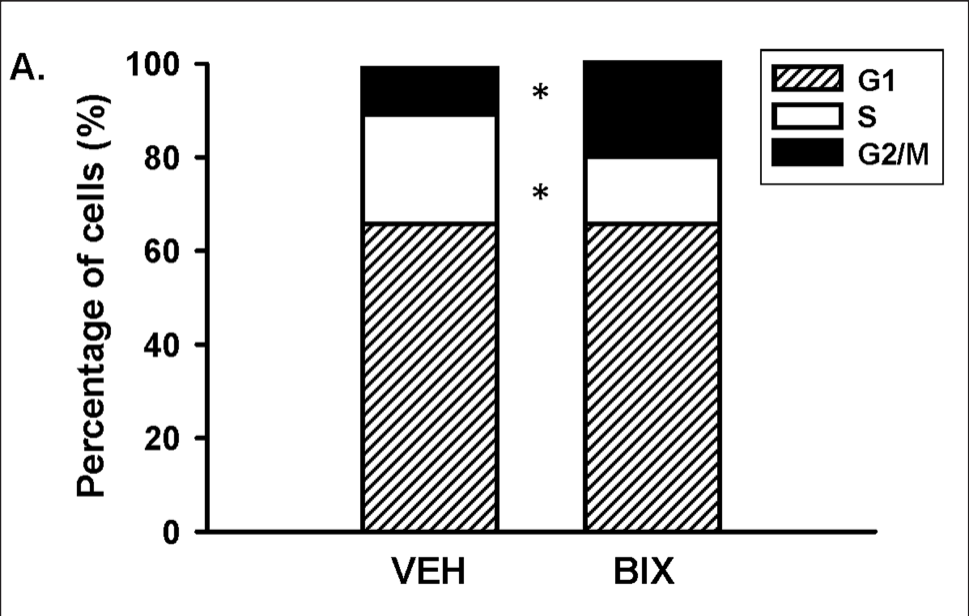
**Inhibition of ERK5 signaling blocks the transition from G2 to M phase of the cell cycle.** NRK-Ctrl cells were incubated with vehicle (DMSO) or BIX-02189 (10 µM) for 16 hours, and cells were fixed for propidium iodide (PI) staining to detect cell cycle progression by flow cytometry. The percentage of cells in each stage of the cell cycle (G1, S, and G2/M) was graphed. n = 4 different experiments. * P < 0.05 significant difference of each specific cell cycle stage between vehicle- versus BIX-02189 treated cells.

Since ERK5 reduced cell number, we investigated whether a reduction in ERK5 signaling would control cell cycle progression by incubating the NRK-Ctrl cells with either BIX02189 (10 μM) or vehicle (DMSO) (Figure 4). ERK5 has previously been shown to promote the transition from G2 through the M phase of the cell cycle [30,31], but its role to regulate the G1/S phases has conflicting reports in the literature [32,33,34,35,36]. As shown in Figure 4, we determined that ERK5 inhibition resulted in a significant increase (20.4 ± 4.2%; P < 0.05; n = 4) in the percentage of cells at the G2/M transition state compared to vehicle-treated NRK-Ctrl cells (9.8 ± 0.5%; n = 4), and there was a concomitantly reduced percentage of S-phase cells in the presence of BIX02189 (12.6 ± 3.4%; n = 4) compared to vehicle-treatment (22.9 ± 1.0%; n = 4). No significant change in the G1 phase was calculated between the BIX02189- (65.7 ± 1.8%; n = 4) versus vehicle-treated cells (66.0 ± 2.4%). The increased percentage of cells in the G2/M transition state has been shown to be associated with the activation of apoptotic signaling [5,37]. Consistent with this finding, there was a robust increase in the appearance of cleaved caspase-3 (Figure 2G and 2H), which is a common marker for apoptosis, in the AGS3-deficient NRK cells compared to the normal NRK-Ctrl cells.

**Figure 5:**
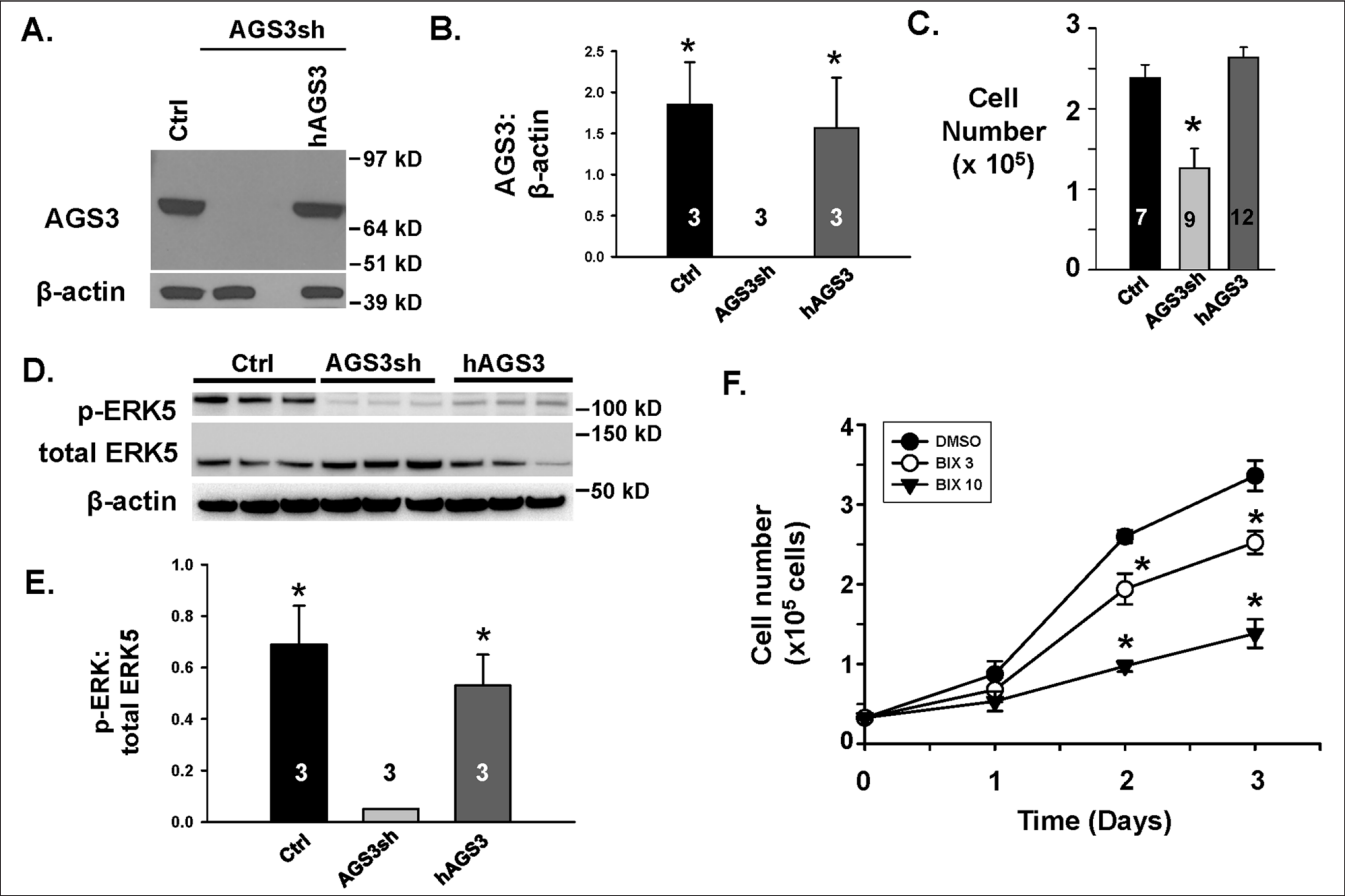
**mTOR phosphorylation status following a reduction in endogenous levels of AGS3.** (A) NRK cells were transduced with the following lentiviral vectors: 1) control (Ctrl); 2) two distinct AGS3-specific shRNA (AGS3sh); or a combination of the AGS3-specific shRNA with a human AGS3cDNA (hAGS3). A representation Western blot is shown to demonstrate AGS3 protein levels. β-actin was used as a loading control. n = 3 different transduced NRK cell lines were generated to perform the cell culture studies in Figures 5 and 6. (B) Densitometry of the AGS3 bands was graphed relative to β-actin. * P < 0.01 significant difference between the NRK-AGS3sh cells. (C) NRK-Ctrl, NRK-AHS3sh, and hAGS3 cells were counted by hemocytometry after 48 hours. n = number of cell counting experiments. * P < 0.05 significant difference between NRK-AGS3sh versus the other two groups. (D) Western blots were performed for p-ERK5 and total ERK5 in NRK-Ctrl and NRK-AGS3sh cells to confirm that recovery of ERK5 activity could be detected following over-expression of human AGS3 in the NRK-AGSsh cells. β-actin was used as a loading control. n = 3 different cell lines tested per genetic modification. Protein standards are shown on the right (in kD) for each blot image. (E) Densitometry of the p-ERK5 and total ERK5 bands were measured and graphed as a ratio. * P < 0.05 significant difference between the NRK-AGS3sh cells. (F) NRK-hAGS3 cells were counted in the presence of either vehicle (DMSO; ●), or two different doses of a selective ERK5/BMK1 inhibitor, BIX02189, at 3 (¡) and 10 µM (▼). *P < 0.05 significant difference between vehicle (DMSO)-treated group. n = 3 experiments per time point.

To rescue the decreased endogenous levels of AGS3 protein, lentiviral vectors encoding human AGS3 were used to transduce the NRK-AGSsh cells to generate a new line, NRK-hAGS3. As shown in Figure 5A and 5B, the human AGS3 protein levels were detected at nearly the same level as the NRK-Ctrl cells expressing scrambled shRNA. Moreover, the cell counts in NRK-hAGS3 cells returned to the same level as the NRK-Ctrl (Figure 5C), and the pattern of cell growth was similar between the NRK-Ctrl and NRK-hAGS3 (Figure 3 compared to Figure 5F). Both NRK-Ctrl and NRK-hAGS3 cell numbers remained significantly higher than the NRK-AGS3sh cells after 48 hours (Figure 5C). Re-expression of human AGS3 in the NRK-AGS3sh cells was able to partially recover the ERK5 activation (Figure 5D and 5E), and treatment with BIX02189 was able to dose-dependently decrease cell number (Figure 5F) similar to our experiments using NRK-Ctrl cells (Figure 3).

**Figure 6:**
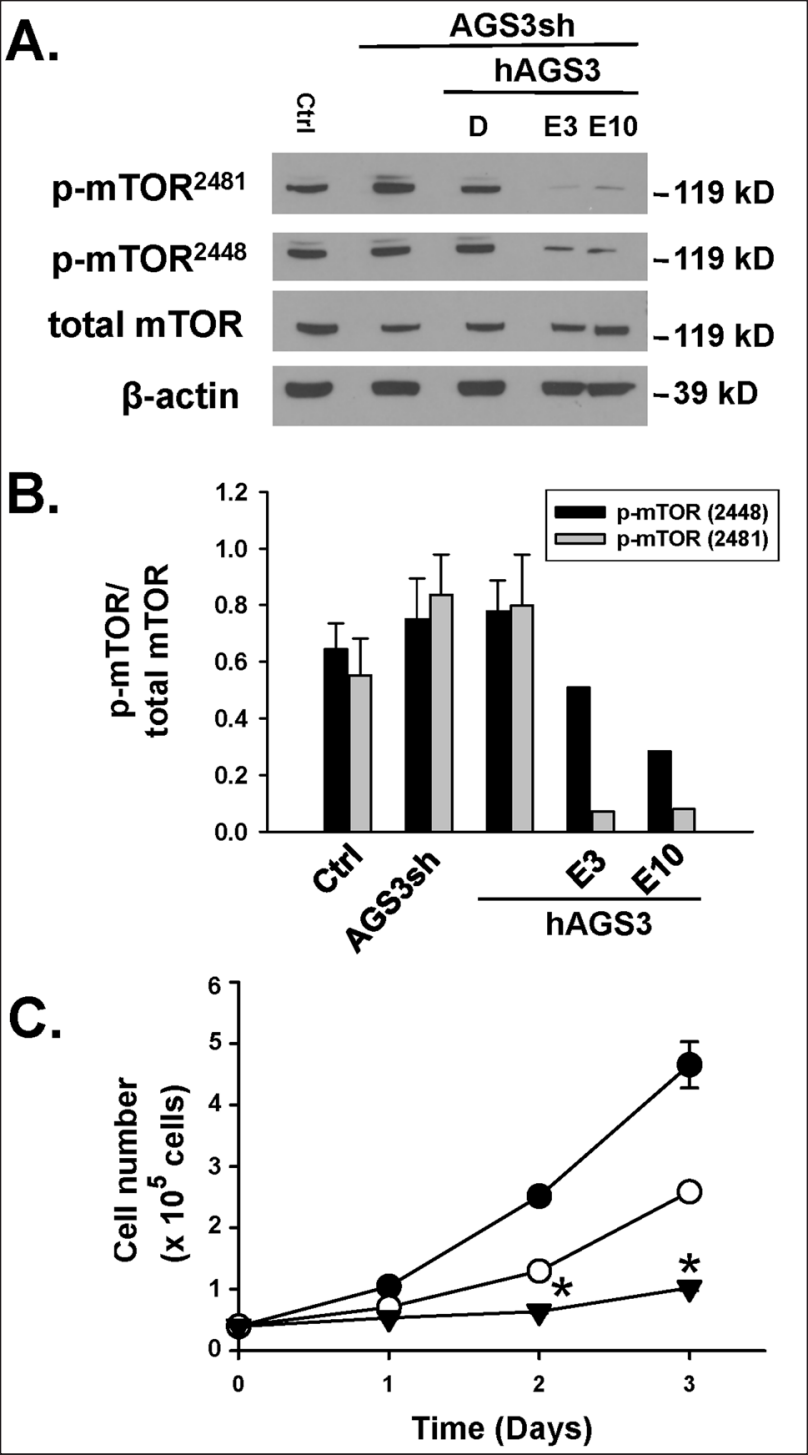
**Lack of a change in mTOR phosphorylation following a reduction in endogenous levels of AGS3.** (A) The same genetically modified cells described in Figure 5 were used to detect the phosphorylation state of mTOR at Ser2448 or Ser2481 by Western blot analysis. In two separate groups of cells, everolimus at 3 and 10 nM was applied for a 24 hr period to confirm as a positive control that mTOR phosphorylation is reduced. β-actin was used as a loading control. Protein standards are shown on the right (in kD) for each blot image. (B) Densitometry of the bands was determined using NIH ImageJ software, and ratios of the phospho-to-total bands were calculated and graphed. (C) NRK-hAGS3 cells were counted by hemocytometry over a 72 hr period in the presence of vehicle (DMSO; ●) or two different doses of a selective mTOR inhibitor, everolimus, at 3 (○) and 10 nM (▼). n = 2–3 experiments per time point. * P < 0.05 significant difference between control versus Everolimus treatment.

**Effect of AGS3 protein depletion on mammalian target of rapamycin (mTOR) activity.** Reduced levels of endogenously expressed AGS3 protein did not alter the phosphorylation status of mTOR at either Ser2448 or Ser2481 compared to NRK-Ctrl or NRK-Ctrl over-expressing human AGS3 protein (NRK-hAGS3; Figure 6A and 6B). Total mTOR protein was not changed regardless of the AGS3 protein levels in the NRK cells.

A selective inhibitor of mTOR, everolimus, was topically added to the NRK at multiple doses (3 and 10 nM) to demonstrate that reduced phosphorylation was associated with decreased cell numbers (Figure 6C). Everolimus markedly reduced both mTOR phosphorylation at Ser2448 and Ser2481 in two separate experiments (Figure 6B), and this was associated with significant reductions in cell number in a dose-dependent manner (Figure 6C).

Similarly, reducing the AGS3 protein levels did not affect the Akt phosphorylation status in NRK cells (unpublished observations). Moreover, incubation of the NRK-hAGS3 cells with a PI3K inhibitor, wortmannin, did not affect cell number (unpublished observations).

In all, these sets of experiments demonstrated that the reduced cell number due to decreased levels of endogenous AGS3 protein was not associated with changes in mTOR activation.

## Discussion

Renal epithelial cells play a critical role in number of biological processes from electrolyte and fluid homeostasis, acid-base balance, hormone secretion, blood pressure regulation and cell volume changes. During the recovery of the kidney following an injury stimulus, the renal epithelial cells can alter their cellular dynamics by either actively undergoing hyperplasia or hypertrophy to maintain their normal level of function. Recent studies in our lab demonstrated an important role for AGS3 as an accessory protein that is involved in the process of epithelial cell repair during acute kidney injury [15] or polycystic kidney disease [12].

Functionally, the GPR-motifs in the C-terminal region of AGS3 act to dock and stabilize up to 4 inactive forms of Gα_i_-GDP subunits. Intuitively, the scavenging of Gα_i_ subunits may directly affect the net production of cAMP, since it is well known that Gα_i_ subunits inhibit adenylyl cyclase activity [18,19]. However, changes in AGS3 expression either has no effect [7,14] or increase the production of steady-state cellular levels of cAMP [18]. The differences in the ability to control adenylyl cyclase activity may be dependent upon the isoform of adenylyl cyclase [5,18], cell type [5,7,14,18], type of G-protein coupled receptor [5,7,38], and/or the duration of the receptor activation leading to desensitization of the adenylyl cyclase system [5]. Moreover, the activation of cAMP regulation may not be directly due to Gα_i_ sequestration, but rather through an indirect action to prevent the re-association with free Gβγ dimers [18]. In many published studies, including the initial identification of Group II AGS proteins, the free Gβγ subunits were the primary activator of AGS3-mediated signaling [2,3,12,14,15,18,20,21,39].

Similar to the findings in the current study, previous studies in our lab have demonstrated that reductions in AGS3 protein levels in renal epithelial cells lead to decreased cell numbers. In addition, we have shown that the growth of NRK epithelial cells was sensitive to Gβγ scavengers [14,15,22] or inhibitors [15,22]. Since Gβγ-mediated signaling has been shown to control mitogen-activated protein kinases (MAPKs), which are well recognized as regulators of pro-survival and proliferative pathways, we initially evaluated whether a reduction in the endogenous expression of AGS3 protein and the associated decrease in cell number were attributed to an interaction with one or more of the MAPKs.

MAPKs are a family of proteins, which are subdivided by their structural similarity, upstream activators, and substrate specificity, and include ERK1/2, SAPK/JNK, p38 MAPK and ERK5 [25,26]. In our study, the activation state of ERK1/2 and p38 MAPK was not changed regardless of the protein expression levels of AGS3. These findings are consistent with recent findings by Branham-O’Connor *et al.* [11] who used immune cells from G-protein signaling modulator 1 (*Gpsm1*)-null mice. In that study, the active form of ERK1/2 under steady-state conditions in the immune cells with or without the expression of AGS3 was similarly low [11]. In response to a chemokine challenge, however, the loss of AGS3 in the immune cells blunted the activation of both ERK1/2 and Akt compared to wild-type AGS3-expressing immune cells [11]. In this case, AGS3 may play a more prominent role in the control of dynamically active signaling pathways through a close association with a membrane-bound receptor, rather than under steady state conditions within the cytoplasm. The proximity of a nearby GPCR may actively promote the dissociation of heterotrimeric G-protein subunits following agonist binding. This would readily enable AGS3 to interact with Gα subunits free from its canonical binding partner, Gβγ, and modulate downstream signal processing. Increased evidence has emerged that AGS3 can directly interact with other proteins to promote discrete localization to either the plasma membrane through its interactions with Gα_i_ proteins [14], G-protein coupled receptor complexes [6,40], or to the base of cilia [21].

Regardless, knockdown of AGS3 protein did significantly reduce the basal activation of ERK5 compared to control NRK cells, which was associated with reduced cell number. Similarly, selective inhibition of ERK5 using BIX02189 resulted in a dose-dependent reduction in cell number compared to control NRK cells expressing endogenous AGS3 or NRK-hAGS3 cells expressing human AGS3 protein. Cell cycle analysis demonstrated an increased percentage of cells within the G2/M transition state in the presence of BIX02189, which was consistent with a previous study using HeLa cells [30]. However, ERK5 may not specifically control entry into the G2/M transition state, but rather other phases of the cell cycle. Regardless of whether ERK5 activation promotes [32,34], inhibits [35], or has no effect [36] on G1/S transition of the cell cycle, it is well recognized that slowing the transition to mitosis from the G2 phase promotes apoptosis [41,42,43]. Our findings were consistent with this phenomenon, since we observed that increased cleaved caspase-3, a classic marker for apoptosis, was associated with a higher percentage of cells at the G2/M transition state. This provided a mechanism by which the AGS3-deficient NRK cells, which have reduced ERK5 phosphorylation, fail to thrive unlike the normal AGS3 expressing NRK cells.

Since the blockade of ERK5 signaling did not entirely prevent cell growth, we investigated other potential signaling pathways that may be regulated by AGS3. One primary candidate was mammalian target of rapamycin (mTOR), which is a key regulator of cell survival by controlling autophagic progression during states of cellular stress. Groves *et al*. [44] observed that increased or decreased levels of AGS3 expression were associated with an increased steady state levels of LC3II, which is a classic marker of autophagic activation in 293T and HeLa cells. In the presence of rapamycin, an mTOR inhibitor, the basal levels of LC3II were modestly increased regardless of the endogenous expression of AGS3. Conversely, Vural *et al*. [16] noted that neither an immortalized macrophage cell line nor primary isolated macrophages isolated from *Gpsm1*^-/-^ mice exhibited any difference in LC3 cleavage or LC3II expression compared to wild-type cells even in the presence of rapamycin. Although there may be cell-type specific effects that exert an influence on mTOR-dependent signaling by AGS3, the mechanism by which AGS3-mTOR interact with each other remains to be fully described. In our study, we were unable to detect any change in the phosphorylation status of mTOR regardless of the endogenous levels of AGS3 in the NRK cells. This was consistent with the findings by Groves *et al*. [44] who mentioned that the activity of S6 kinase 1, a downstream target of mTOR, was not significantly affected in the absence of AGS3 expression. However, in the presence of mTOR inhibitor, rapamycin, the phosphorylation state of AGS3 was markedly impacted in the GoLoco/GPR domains and reduced the cleavage products of LC3 [44]. A previous study showed that the phosphorylation of the GPR motif reduced the ability of AGS3-GPR to associate with Gα_i_ [27], but the mechanism by which a loss in GPR phosphorylation blocks the progression of autophagy to control cell viability remains to be determined.

One caveat regarding the study of signal transduction pathways using genetically modified cells is that changes in signal processing cannot be measured in real-time, but rather in cells that have likely compensated for the gain or loss of a protein of interest. In our case, cell lines were generated to exhibit reduced protein levels of AGS3. Depending upon the counter-regulatory measures that were enacted by the cell to regain normal function, the signaling pathways were analyzed under these conditions. Because of this limitation, the changes in the signaling pathways on ERK5 may not be directly attributed to a change in AGS3 protein function. Future studies will need to be performed using pharmacological inhibitors, which are not currently available, targeted to AGS3 or possibly to the GPR motifs in the C-terminus.

**Perspective on the role of AGS3 in renal injury and disease.** AGS3 has been previously identified in actively proliferating tubular epithelial cells following renal ischemia-reperfusion injury [13,15] and polycystic kidney disease [12]. In AGS3-deficient mice, tubular epithelial cell recovery was attenuated following ischemia-reperfusion injury [15], which would be expected if AGS3-dependent proliferation was blocked during the regeneration phase. In polycystic kidney disease, however, genetic removal of AGS3 resulted in an unexpected acceleration of cystic disease progression [12]. From these studies, it would suggest that AGS3 may control alternate modes of signaling activity independent of proliferation within damaged renal tubular epithelial cells. The present findings by which AGS3 controls apoptotic signaling through an effect on the cell cycle may provide a novel mode of action that connects the observed phenotypes in the kidney during epithelial cell regeneration following acute kidney injury [13,15] and polycystic kidney disease (PKD) [12]. Alterations in AGS3 protein levels may modulate the homeostatic balance between proliferative and apoptotic signaling pathways through its control within interphase of the cell cycle, particularly at the G2/M transition state. Since blockade of apoptosis promoted recovery of the kidney following ischemia-reperfusion injury and attenuated cystic disease progression [45,46], the biological effects exhibited in the kidney due to a loss of AGS3 expression or Gβγ activity [12,15,22] would be consistent with a role for AGS3 to regulate apoptosis. Further studies are needed to determine whether there is any direct relationship between AGS3 and Gβγ activity with ERK5 signaling to control the cell cycle and apoptosis in renal tubular epithelial cells during various state of tubular injury and/or damage.

In summary, reduced levels of AGS3 were associated with lower cell numbers, reduced activity of ERK5, increased accumulation at G2/M transition state, and increased apoptotic signaling. Other MAPK effectors, ERK1/2 or p38 MAPK, or other pro-survival pathways, such as mTOR, appeared to be unaffected by the changes in AGS3 protein expression. Our findings suggest that AGS3-mediated signaling may vary depending upon the cell type being investigated, but elucidate new signaling cascades associated with ERK5 activation in renal epithelial cells.

## Competing Interests

The authors declare that they have no competing interests.
